# Study protocol for the randomised controlled trial: Ketamine augmentation of ECT to improve outcomes in depression (Ketamine-ECT study)

**DOI:** 10.1186/s12888-015-0641-4

**Published:** 2015-10-21

**Authors:** Liam Trevithick, R. Hamish McAllister-Williams, Andrew Blamire, Tim Branton, Ross Clark, Darragh Downey, Graham Dunn, Andrew Easton, Rebecca Elliott, Clare Ellwell, Katherine Hayden, Fiona Holland, Salman Karim, Jo Lowe, Colleen Loo, Rajesh Nair, Timothy Oakley, Antony Prakash, Parveen K Sharma, Stephen R. Williams, Ian M. Anderson

**Affiliations:** Institute of Neuroscience, Newcastle University, Newcastle upon Tyne, UK; Northumberland Tyne and Wear NHS Foundation Trust, Newcastle upon Tyne, UK; Institute of Cellular Medicine, Newcastle University, Newcastle upon Tyne, UK; Leeds and York Partnership NHS Foundation Trust, Leeds, UK; Central Manchester University Hospitals NHS Foundation Trust, Manchester, UK; Neuroscience and Psychiatry Unit, The University of Manchester and Manchester Academic Health Science Centre, Room G809, Stopford Building, Oxford Road, Manchester, M13 9PT UK; Biostatistics Group, The University of Manchester, Manchester, UK; Alpha Hospital, Sheffield, UK; Biomedical Optics Research Laboratory, University College London, London, UK; Pennine Care NHS Foundation Trust, Stockport, UK; Lancashire Care NHS Foundation Trust/ The University of Manchester, Preston, UK; School of Psychiatry, University of New South Wales, Sydney, Australia; Tees, Esk and Wear Valley NTW NHS Foundation Trust, Darlington, UK; Derbyshire Healthcare NHS Foundation Trust, Derby, UK; Manchester Mental Health and Social Care Trust, Manchester, UK; Imaging Science and Biomedical Imaging Institute, The University of Manchester, Manchester, UK

**Keywords:** Ketamine, Major Depressive Episode, Electroconvulsive therapy, Memory, Cognition, Efficacy

## Abstract

**Background:**

There is a robust empirical evidence base supporting the acute efficacy of electroconvulsive therapy (ECT) for severe and treatment resistant depression. However, a major limitation, probably contributing to its declining use, is that ECT is associated with impairment in cognition, notably in anterograde and retrograde memory and executive function. Preclinical and preliminary human data suggests that ketamine, used either as the sole anaesthetic agent or in addition to other anaesthetics, may reduce or prevent cognitive impairment following ECT. A putative hypothesis is that ketamine, through antagonising glutamate receptors, protects from excess excitatory neurotransmitter stimulation during ECT. The primary aim of the ketamine-ECT study is to investigate whether adjunctive ketamine can attenuate the cognitive impairment caused by ECT. Its secondary aim is to examine if ketamine increases the speed of clinical improvement with ECT.

**Methods/Design:**

The ketamine ECT study is a multi-site randomised, placebo-controlled, double blind trial. It was originally planned to recruit 160 moderately to severely depressed patients who had been clinically prescribed ECT. This recruitment target was subsequently revised to 100 patients due to recruitment difficulties. Patients will be randomly allocated on a 1:1 basis to receive either adjunctive ketamine or saline in addition to standard anaesthesia for ECT. The primary neuropsychological outcome measure is anterograde verbal memory (Hopkins Verbal Learning Test-Revised delayed recall task) after 4 ECT treatments. Secondary cognitive outcomes include verbal fluency, autobiographical memory, visuospatial memory and digit span. Efficacy is assessed using observer and self-report efficacy measures of depressive symptomatology. The effects of ECT and ketamine on cortical activity during cognitive tasks will be studied in a sub-sample using functional near-infrared spectroscopy (fNIRS).

**Discussion:**

The ketamine-ECT study aims to establish whether or not adjunctive ketamine used together with standard anaesthesia for ECT will significantly reduce the adverse cognitive effects observed after ECT. Potential efficacy benefits of increased speed of symptom improvement and a reduction in the number of ECT treatments required will also be assessed, as will safety and tolerability of adjunctive ketamine. This study will provide important evidence as to whether adjunctive ketamine is routinely indicated for ECT given for depression in routine NHS clinical practice.

**Trial Registration:**

Current Controlled Trials: ISRCTN14689382 (assigned 30/07/2012); EudraCT Number: 2011-005476-41

## Background

Depression is a leading cause of disability worldwide with unipolar depression ranked 9^th^ in the world and 3^rd^ in Europe amongst causes of health-related disability 2000–2012 [1] and at any one time around 3 % of the UK population meet the criteria for major depression [2]. Effective treatment remains a major problem in depression; in the largest study to date examining patient outcomes in major depression, the STAR*D study, only a quarter to a third of patients remitted after the first antidepressant and a about third had still failed to remit after 4 drug interventions [3].

The National Institute for Health and Clinical Excellence (NICE) recommends electroconvulsive therapy (ECT) as a treatment option for patients with severe depression that is life threatening or those with moderate or severe depression who have not responded to multiple drug treatments and psychological treatment [4]. ECT has been demonstrated to have greater efficacy than pharmacotherapy (effect size, ES -0.8) [5] and achieves remission rates of about 50 % in patients who have failed to respond to drug treatments [6]. It has equal efficacy in both unipolar and bipolar depressed patients [7].

Despite the robust evidence base demonstrating the efficacy of ECT, the number of patients being treated with it has fallen dramatically in recent decades. In England over a 3 month period in 2006, an estimated 1250 patients received ECT compared with over double that number in 1999 [8]. The reduction in ECT usage may be a consequence of concerns about a poor riskbenefit balance due to adverse cognitive side effects [9]. ECT treatment is associated with significant objective impairments in cognitive function, the largest effect being on verbal delayed recall, but also with significant impairment in all tests of anterograde memory, executive function and processing speed [10]. Following the end of ECT treatment there is a rapid reversal of deficits, with moderate to large improvements above baseline in most measures after 1–2 weeks [10]. However, one study showed enduring deficits in spatial recognition memory one month after end of treatment [11]. There is uncertainty as to the magnitude and persistence of retrograde amnesia following ECT [12], though a systematic review suggested that 29–55 % of patients reported persistent and often distressing loss of important past memories after ECT [13]. It has been reported that there is an association between subjective memory impairment and objective autobiographical memory impairment immediately after ECT course as well as 6 months later [14]. A survey across multiple clinical hospitals suggested that the severity, incidence and persistence of memory and cognitive dysfunction after ECT was related to treatment approach [15].

The precise mechanisms underlying the effects of ECT are not fully understood, but altered synaptic functioning in neural circuits involved in mood and cognition is thought to play a key role [16]. Glutamate, an amino acid neurotransmitter with a role in both neuroplasticity and excitotoxicity, acts on a variety of receptors in the brain, most commonly N-methyl-d-aspartate (NMDA) and α-amino-3-hydroxy-5-methylisoxazole-4-propionic acid (AMPA) receptors [17]. Preclinical evidence supports a role for glutamate in mood regulation and in the action of antidepressant treatments [17, 18]; in particular decreased NMDA-mediated, and increased AMPA-mediated neurotransmission has been proposed [18]. Studies in depressed patients have found decreased frontal cortical measures of glutamate measured in vivo by magnetic resonance spectroscopy (MRS) which normalises with effective treatment [17]. There are similar findings with ECT; pre-treatment glutamate and glutamate-related concentrations have been found in dorsolateral prefrontal cortex [19] and anterior cingulate cortex [20, 21] which either normalised after ECT [19, 21] or predicted treatment response [20].

Glutamate also has a central role in cognition especially learning and memory, through its role in synaptic plasticity and the signalling pathway involved in long-term potentiation in the hippocampus [22]. It has been proposed that the memory impairment occurring with ECT is a consequence of indiscriminate activation/saturation of glutamate receptors at the time of the seizure leading to a disruption of hippocampal plasticity involved in memory [23]. Autobiographical memory impairment may occur through disruption of the reconsolidation of ‘fragile’ reactivated or recalled memories [24].

Ketamine is a dissociative anaesthetic, analgesic and psychotomimetic which inhibits NMDA receptors but also stimulates glutamate release and potentiates activity at non-NMDA receptors such as AMPA receptors [25]. Recent studies have shown a rapid antidepressant effect after a single dose of intravenous ketamine alone or as an adjunct to antidepressants in both unipolar and bipolar depression [26]. Repeated administration maintains improvement but relapse occurs rapidly on stopping treatment [27]. Side effects of ketamine treatment include acute anxiety, increased mood instability, vomiting, and vasovagal episodes, dissociation, dry mouth, tachycardia and increased blood pressure [27], though more severe cardiovascular side effects are associated with higher ketamine doses (0.8–1.0 mg/kg) [26].

Given acutely ketamine can cause cognitive impairment [28], particularly in manipulating information in working memory and encoding into episodic memory. However, preliminary human data from retrospective/non-randomised studies suggested that ketamine anaesthesia improves reorientation [29, 30] and word recall [31] after ECT, and also may result in more rapid clinical response [32]. Subsequent small randomised controlled trials (RCTs) have produced mixed results with studies suggesting impaired reorientation [33, 34] and improved [35] or unchanged [33] Mini Mental State Examination (MMSE) scores after ketamine. A further study found that ketamine augmentation produced no reduction in cognitive impairment after ultra-brief right unilateral stimulation on a range of tests [36]; however this technique is associated with minimal cognitive impairment compared with standard bilateral ECT which limits interpretation. Two recent meta-analyses looking at efficacy, and including the same 4 placebo-controlled RCTs in which ketamine has been combined with ECT, have reached different conclusions. When continuous measures were pooled a moderate to large advantage for ketamine was reported early in the ECT treatment course [26], but no benefit was found when end-of-treatment response and remission were meta-analysed [37]. The latter meta-analysis also reported higher rates of confusion/disorientation/prolonged delirium with ketamine. At present the small heterogeneous studies of ketamine augmentation of ECT emphasise the need for further larger scale trials.

Depression is associated with impaired frontal cortical metabolism and function [38, 39] and treatment with antidepressant drugs leads to their normalisation [38, 40]. In contrast ECT is associated acutely with a further decrease in frontal cortex metabolism [41], and one hypothesis is that the cognitive deficits seen with ECT may relate to these frontal effects [42]. If ketamine prevents cognitive deficits with ECT then a test of this hypothesis is whether ketamine prevents ECT-induced suppression of frontal cortical function.

### Aims and Hypotheses

The primary aim of the Ketamine-ECT study is to investigate the effect of adjunctive ketamine on cognitive dysfunction caused by ECT using an RCT design with the hypothesis that ketamine, compared with saline treatment, given together with standard anaesthesia will reduce ECT-induced cognitive impairment. Secondary aims are to 1) investigate the efficacy, tolerability and acceptability of adjunctive ketamine (we hypothesise that ketamine treatment will lead to a more rapid improvement in depressive symptoms with fewer ECT treatments needed to achieve remission) and 2) to identify whether ketamine modifies ECT’s effect on frontal cortical function using functional near infrared spectroscopy (fNIRS) during performance of cognitive tasks.

## Methods/Design

### Study Design

The Ketamine-ECT study is a randomised, parallel group, placebo-controlled, double blind trial of ketamine (0.5 mg/kg), compared to saline, added to standard anaesthetic induction, in depressed patients who have been prescribed ECT. Both participants and researchers will be blinded to the treatment arm to which patients are allocated. In the interests of patient safety, the anaesthetic team will be aware of study treatment allocation, with this information disclosed to other clinical or research team members only if safety concerns arise.

### Study Population

The study will recruit hospital in-patients and out-patients with moderate to severe depression who have been prescribed ECT by their treating psychiatrist and have been determined fit for ECT and ketamine administration by an anaesthetist. All participants will provide written informed consent. The original aim was to recruit 160 patients, however due to slow recruitment a revised target of 100 patients, and refinement of the primary outcome measure, together with a study extension, was agreed between the study team, Data Monitoring and Ethics Committee (DMEC) and Trial Steering Committee (TSC), Research Ethics Committee, the Medicines and Healthcare products Regulatory Authority (MHRA), the funder and the sponsor. The recruitment target for a sub-population drawn from those taking part in the main RCT to undergo mechanistic studies was originally 100 (revised to 20 due to slow recruitment) together with 50 matched healthy volunteers recruited for neuropsychological and fNIRS baseline testing comparisons.

#### Patient Inclusion criteria

- Male or Female aged 18 years and above prescribed ECT by their treating psychiatrist.
- Current Diagnostic and Statistical Manual of Mental Disorders-Fourth Edition (DSM-IV) diagnosis of a major depressive episode, moderate or severe, as part of unipolar or bipolar disorder, diagnosed using the Mini International Neuropsychiatric Interview (MINI) [43].
- American Society of Anesthesiologists score of 1, 2 or stable 3 (excluding mental health considerations in the scoring), and judged as suitable to receive ketamine by an anaesthetist.
- Verbal IQ [44] equivalent to 85 or greater and sufficiently fluent in English to validly complete neuropsychological testing.
- Capacity to provide written informed consent to take part in the study.

#### Patient Exclusion criteria

- DSM-IV diagnosis of a primary psychotic or schizoaffective disorder, current primary obsessive compulsive disorder or anorexia nervosa.
- History of drug or alcohol dependence (DSM-IV criteria) within the last year.
- ECT in last 3 months or has previously received ECT in the current trial.
- Known hypersensitivity or contraindication to ketamine or excipients in the injection, including significant cardiovascular disease, uncontrolled hypertension, glaucoma, cirrhosis or abnormal liver function or liver disease.
- Known hypersensitivity or contraindication to concomitant medications used for ECT including propofol or suxamethonium or excipients in the injections.
- Evidence of organic brain disease including dementia, neurological illness or injury, or medical illness which may significantly affect neuropsychological function.
- Detained under the Mental Health Act (1983 as amended 2007).
- Pregnancy, or at risk of pregnancy and not taking adequate contraception, breastfeeding.
- Score of less than 24 on the MMSE [45].

Psychotropic medication-free healthy volunteers without a personal or family history of major psychiatric illness or significant medical illness will also be recruited. They will be aged over 18 years or over and will be matched as a group with patients for age and sex.

### Recruitment

Patient recruitment was originally planned for 6 NHS Trusts in the North of England. This was subsequently increased to 7 Trusts, with 11 ECT suites. Recruitment for the fNIRS mechanistic sub-study, including healthy volunteers, will be based in two of the centres where the equipment will be based (Manchester and Newcastle), with opportunity to recruit from other sites when practically feasible to transport the equipment to patients.

### Intervention

ECT and anaesthetic treatment protocols will be determined by the local ECT services but is required to be consistent with requirements given in the Royal College of Psychiatrists’ ECT handbook [46]; ECT being given twice a week. The anaesthetic induction agent of choice is propofol, with thiopental permissible as an alternative. For safety reasons etomidate cannot be used to avoid the potential for an interaction with ketamine excessively elevating blood pressure. Oral psychotropic medication continued by the patient’s treating clinical team will remain unchanged where possible for at least the first 4 ECT treatments, and ideally until end of ECT. The goal of ECT is to treat patients to remission (Montgomery-Åsberg Depression Rating Scale, MADRS ≤10) in accordance with NICE guidelines [4]. The final decision to end ECT will rest with the treating clinical team in consultation with the patient and the local ECT treatment team. Following recruitment, and before their first ECT treatment, patients will be randomised in a 1:1 ratio to receive ketamine 0.5 mg/kg or saline as a slow bolus before the anaesthetic induction agent. Randomisation will be by permuted block randomisation, stratified by NHS Trust, carried out by the Manchester Academic Health Science Centre Clinical Trials Unit (http://www.mahsc-ctu.co.uk/). Randomisation will occur by telephone and allocation communicated securely to each Trust Pharmacy which will securely over-package the study drug to conceal allocation. On the day of each treatment the anaesthetist will open the package and prepare the injection away from the ECT team and patient to maintain their blinding. Patients will receive the same allocated study medication throughout their course of ECT.

### Outcome measures

**Fig. 1:**
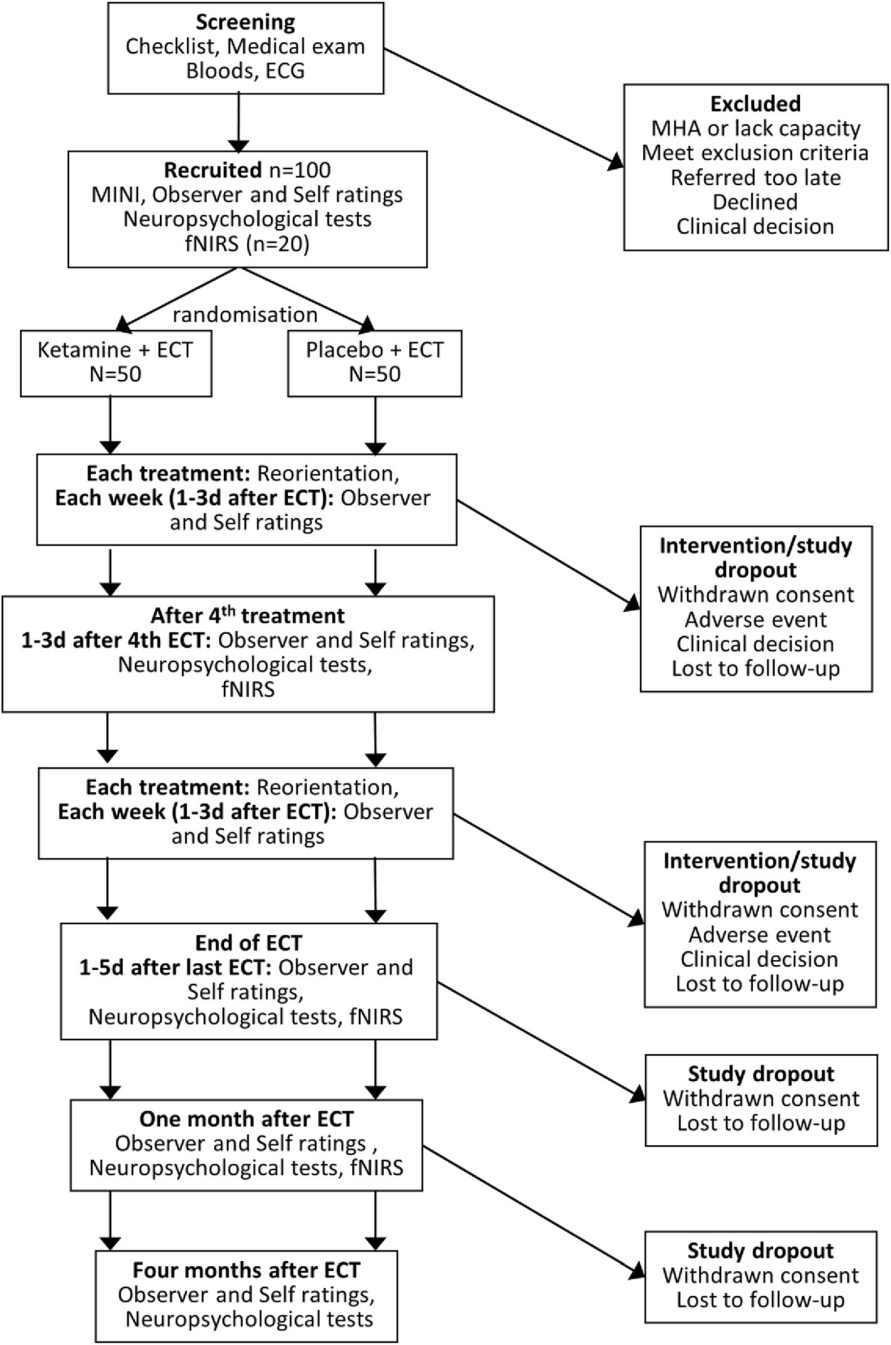
Flowchart of schedule of visits. fNIRS: function near infrared spectroscopy; MINI: Mini International Neuropsychiatric Interview; Observer ratings: Breif Psychiatric Rating Scale; Montgomery Åsberg Depression Rating Scale; Clinical Global Impression; Self ratings; EuroQol; Quick Inventory of Depressive Symptomatology-Self Report

All assessments will be undertaken by trained research personnel under the supervision of the clinically trained principle investigators. Figure 1 shows the study schedule for visits and assessments. The primary outcome time point will be after the fourth ECT treatment.

#### Neuropsychological measures

The Hopkins Verbal Learning Test-Revised (HVLT-R) [47] will be used as the primary outcome measure to assess anterograde memory. Secondary measures are described in Table 1. The cognitive assessment battery has been shown in previous research to be sensitive to the effects of ECT [36] and has been chosen to also be short enough in duration to be acceptable to patients.

**Table 1:** Description of neuropsychological and clinical efficacy outcome measures

Abbreviation	Full Name	Description	Reference
Neuropsychological Measures			
HVLT-R	Hopkins Verbal Learning Test – Revised	Anterograde verbal learning and memory assessment	[47]
AMI-SF	Autobiographical Memory Inventory – Short Form	Retrograde assessment of personal memories	[59]
COWAT – letter and category	Controlled Oral Word Association Test	Letter and Category fluency assessment	[60]
MCG – Complex Figure Test	Medical College of Georgia Complex Figure Test	Anterograde visuospatial memory assessment	[61]
Digit Span	Digit Span	Working memory assessment	[62]
GSE-My	Global Self-Evaluation of Memory	Self-reported assessment of memory	[63]
Reorientation	Reorientation	Number of 5 orientation items correct 30 and 60 min after ECT	[64]
Clinical Efficacy Measures			
MADRS	Montgomery-Åsberg Depression Rating Scale	Observer-rated assessment of depressive symptoms	[48]
CAS	Clinical Anxiety Scale	Observer-rated assessment of anxiety symptoms	[65]
BPRS	Brief Psychiatric Rating Scale	Observer-rated assessment of psychiatric symptoms	[66]
CGI-S, CGI-I	Clinical Global Impression – Severity and Improvement	Observer-rated global measure of illness severity and improvement	[67]
QIDS-SR	Quick Inventory Depressive Symptomology – Self Rated	Self-rated measure of depressive symptoms	[68]
EQ-5D	EuroQol	Self-rated measure of health-related quality of life	[69]

#### Clinical efficacy measures

The primary efficacy measure will be change in MADRS score [48] from baseline with secondary outcomes listed in Table 1.

#### Mechanistic measures

Bilateral dorsolateral and ventrolateral prefrontal cortex response to computerised tasks of verbal fluency (for category) and working memory (N-Back) will be measured using fNIRS, a portable brain imaging technique that can be used at the bedside or in a person’s home. This uses the differential light absorption properties of oxyhaemoglobin (HbO) and deoxyhaemoglobin (HbR) for near infrared light when it passes through the scalp, skull and superficial layers of the cortex from a light source placed on the scalp. The change in the light absorption spectra are measured with detectors placed nearby also on the scalp. The technique is analogous to functional magnetic resonance imaging (MRI) using blood oxygen level dependent changes, although it measures both HbO and HbR. It is possible to use fNIRS as a measure of functional changes in cortical blood flow secondary to neuronal activation [49].

In addition, a spatially resolved spectroscopy technique will be used, employing a modification of the diffusion equation of light transport, to provide an absolute measure of tissue oxygenation (the tissue oxygenation index). This index is the absolute percentage of total haemoglobin in the field of view which is oxygenated [50]. Its reliability and validity has yet to be established [51], however it does provide a potential absolute measure with which to assess cerebral tissue metabolism across groups and within subjects [52].

Two purpose built optical tomography systems (Biomedical Optics Research Laboratory, Dept. of Medical Physics and Bioengineering, University College London) provide a 48 channel array for topographical coverage of bilateral dorsolateral and ventrolateral prefrontal cortices.

Originally it had been planned to undertake glutamate magnetic resonance spectroscopy and structural and functional MRI in a subgroup, but this had to be halted when it became clear that insufficient patients would be recruited to allow valid analysis.

### Sample size, power and effect size

#### Original sample size and power calculation

Across the 6 NHS Trusts originally involved in the study, 355 patients received ECT over a 12 month period in 2009/10, the most recent period available when planning the study. The most comparable recent study in the UK [53] found that 41 % of patients receiving ECT were eligible, using similar inclusion/exclusion criteria to ours, and 18 % were randomised (to either ECT or transcranial magnetic stimulation). Given the current study offers adjunctive, rather than alternative treatment, a 22.5 % recruitment rate was predicted. Based on these figures recruitment of 160 patients over a 24 month recruitment period was forecast as feasible, providing 152 patients for primary end point assuming 95 % of patients can be assessed after 4 treatments.

The power calculation was based on a clinically useful benefit of a moderate standardised effect size (ES) for the ketamine-saline difference (0.5–0.6). With a 90 % power, and two-sided *p* = 0.05, based on independent t-tests, we would have been able to detect a standardised ES of 0.51 for full Intention-To-Treat (ITT) and 0.54 for a single primary cognitive outcome based on completer assessments. Using a Bonferroni correction for the originally proposed three measures for the primary cognitive outcome, HVLT-R, COWAT category and AMI-SF (i.e., assuming independence) gives an 80 % power to detect a standardised ES of 0.51 given a two-sided, corrected, *p* = 0.05 for each measure.

The study aimed to recruit 100 patients (50 per treatment group) and 50 matched healthy controls to the mechanistic studies. This would have an 80 % power to detect an ES of 0.5 with a two-sided alpha level of 0.05 between groups and an ES of 0.57 with a two-sided alpha level of 0.05 between treatment arms. A previous fNIRS study in depressed patients [54] found an ES difference from controls of 0.8 in frontal cortex HbO response to a verbal fluency task.

#### Revised Power calculations

Following initial slow recruitment, the revised recruitment target was adjusted to 100 patients with 95 reaching the primary outcome measure point and the primary outcome was changed to the HVLT-R alone. This sample size provides 80 % power to detect an ES of 0.57 (ITT) and 0.58 (completer), two-sided *p* = 0.05. The target for recruitment to the mechanistic studies was decreased to a sample size of 20 patients (10 per treatment arm) and 50 controls. This has only a 21 % power to detect an ES of 0.6 with a two-sided *p* = 0.05 between the two treatment arms (10 per arm). Comparing 20 patients and 50 controls will give an ability to detect an ES of 0.6 between groups with 60 % power and two-sided *p* = 0.05, and a 70 % power to detect the same size of effect of ECT itself with a two-side *p* = 0.05.

### Statistical Analysis

#### Neuropsychological and clinical outcomes

There are no interim analyses planned unless requested for safety reasons by the DMEC. All estimates of efficacy will be based on a modified intention to treat (ITT) analysis including patients who have received at least one ECT treatment (given that this is a study of the effect of ketamine on ECT). The main statistical inference for both neuropsychological and efficacy outcomes will be made after 4 ECT sessions. In addition, the two treatment groups will be compared at the end of acute ECT treatment and 1 and 4 months later. Analysis methods are specified in a detailed Statistical Analysis Plan approved by the DMEC and TSC (available on request). Briefly, for neuropsychological data cross-sectional analysis of covariance (ANCOVA) models (allowing for stratifying variables, age, sex, baseline degree of treatment resistance, electrode placement [bilateral or unilateral] and baseline values of the outcome being studied) will be used to evaluate the effects of treatment allocation. The sensitivity of the findings to missing data will be assessed with inverse probability weighting adjustments. All analyses will involve the use of robust standard errors and associated confidence intervals (allowing for non-normality and constraints in the ranges of some of the cognitive outcomes). Efficacy data will be analysed using a random effects (random intercepts) ANCOVA model with time (in weeks) as a quantitative explanatory variable. The baseline variables will be the same as those for neuropsychological data. An interaction term between time and treatment allocation will also be included to assess the treatment effect.

#### Mechanistic outcomes

fNIRS data will be preprocessed with HOMER2 (http://www.nmr.mgh.harvard.edu/PMI/resources/homer2/home.htm) converting them into HbO and HbR concentration changes after noise and motion artefact correction [55]. Primary analysis will be the region of interest (ROI) covering the key frontal cortical area and consist of the anterior optical channels. Channels will be discarded for very low optical intensity or high motion artefact according to standard protocols. Stimulus blocks will be averaged and the maximum haemodynamic changes determined for both HbO and HbR concentration in a time window based on the expected peak latency of the haemodynamic response. Repeated measures analysis of variance will be performed to compare patients and healthy controls and the effect of ECT and treatment allocation in patients, assigning 0 values to the missing channels within the ROI.

### Status of the Study

The study was registered on 30/07/2012 (ISRCTN14689382) under the public title “Ketamine ECT study”. Clinical Trial Authorisation was given by the Medicines and Healthcare products Regulatory Agency (MHRA: EudraCT: 2011-005476-41). Ethical approval was granted by the North West-Liverpool East Research Ethics Committee (REC Ref No. 12/NW/0021) on 25/01/2012. Recruitment commenced in December 2012 and an extension to the study and to recruitment was granted by the funder in 2014, with the study finishing in late 2015.

## Discussion

The Ketamine ECT study will be one of the largest (even after the reduction in the recruitment target), and one of the very few multicentre, RCTs investigating ECT practice that has been conducted in the UK for decades. Previous research has been inconclusive about whether or not there is a benefit from augmenting ECT with ketamine, both in terms of reducing cognitive impairment, and improving symptomatic outcome or rate of improvement (see Background), and a prospective RCT is required to determine whether this is clinically useful and safe, particularly given some evidence that ketamine might increase the post-treatment recovery time following ECT [37], and the limited information about the risks of repeated ketamine administration in this setting. Further, no trial to date has assessed the neurocognitive effects of augmenting bitemporal ECT with ketamine.

The recognised problem of cognitive impairment with ECT is one of the reasons why it is increasingly being reserved for only the very severest cases of depression, and possibly for an older non-working population, in spite of its efficacy [9]. The numbers of patients receiving ECT in England and Wales has decreased from approximately 20,000 a year in the 1980s [56] to under 5000 in 2006 [8]. The Scottish ECT Audit Network also shows a continuing gradual decline between 2006 and 2013 [57]. If ketamine augmentation can significantly reduce the cognitive impact of ECT, and it is adopted as a standard treatment, this may lead to a re-evaluation of the risk-benefit balance for ECT and potentially make it more accessible to a wider range of patients. A major problem in the treatment of depression is the limited efficacy of current treatments, particularly after insufficient response to initial therapy. This has been highlighted most strikingly by the STAR*D study in the United States [3] which showed that only a quarter to a third of patients remit with initial treatment, and that remission rates fall to a seventh in patient who have failed two or more treatments. ECT is now only used infrequently in this situation in spite of evidence that two thirds of treatment-resistant depressed patients have a good clinical response, and nearly half remit [6, 57]. Protection against the cognitive adverse effects of ECT would potentially make it a more acceptable and accessible therapeutic option for treatment resistant patients. In addition if speed of response is greater with ketamine augmentation, this could reduce the number of ECT treatments required, not only potentially reducing the cognitive impact of treatment, but also making ECT more cost-effective.

RCTs involving ECT face a number of methodological challenges. One difficulty in the assessment and analysis of ECT trials is the lack of a defined number of treatments making up a course of ECT. Some patients respond very rapidly and need only a few treatments, others require prolonged courses. In addition it is not uncommon for individual treatments in a course to be missed due to patient or timetabling factors. Unlike drug treatments, ECT is not normally continued past the point of clinical remission, so that assigning a particular time period, or number of ECTs, for an end of treatment assessment is problematic. The solution adopted in this study is to make the primary assessment point after 4 ECT treatments received. Clinical experience suggests that most patients receive at least 4 treatments (unless they drop out due to adverse effects); at this point cognitive effects of ECT are apparent [58] and most patients will have shown some, if not full, improvement. An effect of ketamine in reducing cognitive impairment, and increasing antidepressant effect, should be detectable at this time point. Secondary time point assessment will attempt to capture the effects at end of ECT and in follow up. The assessment of the cognitive impact of ECT is also challenging as cognitive impairments rapidly improve in the period of time following the last ECT treatment [10]. This means that there is a relatively narrow window of opportunity to assess any cognitive changes. Having the primary endpoint during the course of ECT is helpful in this regard as it constrains the window of assessment to 1-3 days after a treatment.

From the outset, recruitment to the study was anticipated to be challenging due to the narrow recruitment window between the decision to have ECT and the first treatment. In practice recruitment was further reduced by two additional factors. First, the number of patients receiving ECT has been decreasing so that the need to ensure a large enough pool from which to recruit has meant involving a large number of Trusts and ECT suites, each with their own procedural and governance requirements. Additionally, during set-up and the study itself, three ECT suites have closed due to NHS Trust reorganisations, requiring the identification of new sites. The delayed initiation of these new sites reduced capacity and available time to reach full recruitment. Second, the decrease in ECT prescribing has been combined with a trend to treat, on average, more severely ill patients, a higher proportion of whom than expected are ineligible for the study due to detention under the Mental Health Act or lack of capacity to consent. This has restricted our rate of recruitment, nevertheless, the revised recruitment target will still provide power to identify a clinically important effect size on a key cognitive measure, anterograde verbal memory measured by the HVLT-R.

With regard to the mechanistic studies, recruitment has been especially difficult because the clinical sites are more geographically scattered than originally planned, severely limiting the numbers of patients sufficiently close to the two imaging centres for MR imaging. It has raised practical difficulties in transporting the optical imaging equipment for the fNIRS assessments and in being able to do the testing before the first ECT session. In addition the severity of illness of the patients has meant that many are reluctant to undergo additional testing above the neuropsychological and efficacy assessments. This has meant having to discontinue the MR imaging and to severely curtail the recruitment numbers for fNIRS. Nevertheless we will be able to assess the feasibility and usefulness of fNIRS in this population in assessing depression and measuring and predicting the effects of ECT treatment, although the power to detect effects of ketamine augmentation will be very low.

In summary this is an important study which has the potential to alter the practice of ECT and to improve the risk-benefit balance of ECT for patients who need to receive it. If successful it may allow wider access to the most effective known acute treatment for depression. Unfortunately recruitment difficulties as a consequence of the decline in ECT usage, the severity of the illness of patients receiving ECT, and the changes in ECT provision will reduce the power of the study to provide as definitive an answer as originally planned.

## Abbreviations

AMI-SF: Autobiographical memory inventory-short form
AMPA: α-amino-3-hydroxy-5-methylisoxazole-4-propionic acid
ANCOVA: Analysis of covariance
BPRS: Brief psychiatric rating scale
CAS: Clinical anxiety scale
CGI-I: Clinical global impression-improvement
CGI-S: Clinical global impression-severity
COWAT: Controlled oral word association test
DMEC: Data monitoring and ethics committee
DSM-IV: Diagnostic and statistical manual of mental disorders-fourth edition
ECT: Electroconvulsive therapy
ES: Effect size
fNIRS: Functional near-infrared spectroscopy
GSE-My: Global self-evaluation of memory
HbO: Oxyhaemoglobin
HbR: Deoxyhaemoglobin
HVLT-R: Hopkins verbal learning test-revised
MADRS: Montgomery-åsberg depression rating scale
MCG: Medical College of Georgia
MHRA: Medicines and healthcare products regulatory authority
MINI: Mini international neuropsychiatric interview
MMSE: Mini mental state examination
MRI: Magnetic resonance imaging
NICE: National Institute for Health and Clinical Excellence
NMDA: N-methyl-d-aspartate
RCT: Randomised controlled trial
ROI: Region of interest
TSC: Trial Steering Committee

## References

[1] World Health Organisation. Health statistics and information systems: estimates for 2000-2012 - disease burden. http://www.who.int/healthinfo/global_burden_disease/estimates/en/index2.html. 2015. accessed 11-8-2015.

[2] Singleton N, Bumpstead R, O’Brien M, Lee A, Meltzer H (2001). Psychiatric Morbidity Among Adults Living in Private Households, 2000.

[3] Rush AJ, Trivedi MH, Wisniewski SR, Nierenberg AA, Stewart JW, Warden D (2006). Acute and longer-term outcomes in depressed outpatients requiring one or several treatment steps: a STAR*D report. Am J Psychiatry.

[4] National Institute for Health and Clinical Excellence. Clinical Guideline 90. Depression in adults (update): full guideline. http://guidance.nice.org.uk/CG90/Guidance . 2009. accessed 11-8-2015

[5] UK ECT Review Group (2003). Efficacy and safety of electroconvulsive therapy in depressive disorders: a systematic review and meta-analysis. Lancet.

[6] Heijnen WT, Birkenhager TK, Wierdsma AI, van den Broek WW (2010). Antidepressant pharmacotherapy failure and response to subsequent electroconvulsive therapy: a meta-analysis. J Clin Psychopharmacol.

[7] Dierckx B, Heijnen WT, van den Broek WW, Birkenhager TK (2012). Efficacy of electroconvulsive therapy in bipolar versus unipolar major depression: a meta-analysis. Bipolar Disord.

[8] Bickerton D, Worrall A, Chaplin R (2009). Trends in the administration of electroconvulsive therapy in England. Psychiatr Bull.

[9] National Institute for Clinical Excellence. Technology Appraisal 59. Guidance on the use of electroconvulsive therapy. http://guidance.nice.org.uk/TA59/Guidance/pdf/English. 2003. accessed 11-8-2015

[10] Semkovska M, McLoughlin DM (2010). Objective cognitive performance associated with electroconvulsive therapy for depression: a systematic review and meta-analysis. Biol Psychiatry.

[11] Falconer DW, Cleland J, Fielding S, Reid IC (2010). Using the Cambridge Neuropsychological Test Automated Battery (CANTAB) to assess the cognitive impact of electroconvulsive therapy on visual and visuospatial memory. Psychol Med.

[12] Fraser LM, O’Carroll RE, Ebmeier KP (2008). The effect of electroconvulsive therapy on autobiographical memory: a systematic review. J ECT.

[13] Rose D, Fleischmann P, Wykes T, Leese M, Bindman J (2003). Patients’ perspectives on electroconvulsive therapy: systematic review. BMJ.

[14] Brakemeier EL, Berman R, Prudic J, Zwillenberg K, Sackeim HA (2011). Self-evaluation of the cognitive effects of electroconvulsive therapy. J ECT.

[15] Sackeim HA, Prudic J, Fuller R, Keilp J, Lavori PW, Olfson M (2007). The cognitive effects of electroconvulsive therapy in community settings. Neuropsychopharmacology.

[16] Merkl A, Heuser I, Bajbouj M (2009). Antidepressant electroconvulsive therapy: mechanism of action, recent advances and limitations. Exp Neurol.

[17] Mitchell ND, Baker GB (2010). An update on the role of glutamate in the pathophysiology of depression. Acta Psychiatr Scand.

[18] Sanacora G, Zarate CA, Krystal JH, Manji HK (2008). Targeting the glutamatergic system to develop novel, improved therapeutics for mood disorders. Nat Rev Drug Discov.

[19] Michael N, Erfurth A, Ohrmann P, Arolt V, Heindel W, Pfleiderer B (2003). Metabolic changes within the left dorsolateral prefrontal cortex occurring with electroconvulsive therapy in patients with treatment resistant unipolar depression. Psychol Med.

[20] Merkl A, Schubert F, Quante A, Luborzewski A, Brakemeier EL, Grimm S (2011). Abnormal Cingulate and Prefrontal Cortical Neurochemistry in Major Depression After Electroconvulsive Therapy. Biol Psychiatry.

[21] Pfleiderer B, Michael N, Erfurth A, Ohrmann P, Hohmann U, Wolgast M (2003). Effective electroconvulsive therapy reverses glutamate/glutamine deficit in the left anterior cingulum of unipolar depressed patients. Psychiatry Res.

[22] Peng S, Zhang Y, Zhang J, Wang H, Ren B (2011). Glutamate receptors and signal transduction in learning and memory. Mol Biol Rep.

[23] Gregory-Roberts EM, Naismith SL, Cullen KM, Hickie IB (2010). Electroconvulsive therapy-induced persistent retrograde amnesia: could it be minimised by ketamine or other pharmacological approaches?. J Affect Disord.

[24] Alberini CM, Milekic MH, Tronel S (2006). Mechanisms of memory stabilization and de-stabilization. Cell Mol Life Sci.

[25] Maeng S, Zarate CA (2007). The role of glutamate in mood disorders: results from the ketamine in major depression study and the presumed cellular mechanism underlying its antidepressant effects. Curr Psychiatry Rep.

[26] Fond G, Loundou A, Rabu C, Macgregor A, Lancon C, Brittner M (2014). Ketamine administration in depressive disorders: a systematic review and meta-analysis. Psychopharmacology.

[27] Diamond PR, Farmery AD, Atkinson S, Haldar J, Williams N, Cowen PJ (2014). Ketamine infusions for treatment resistant depression: a series of 28 patients treated weekly or twice weekly in an ECT clinic. J Psychopharmacol.

[28] Morgan CJ, Curran HV (2006). Acute and chronic effects of ketamine upon human memory: a review. Psychopharmacology (Berl).

[29] Krystal AD, Weiner RD, Dean MD, Lindahl VH, Tramontozzi LA, Falcone G (2003). Comparison of seizure duration, ictal EEG, and cognitive effects of ketamine and methohexital anesthesia with ECT. J Neuropsychiatry Clin Neurosci.

[30] Kranaster L, Kammerer-Ciernioch J, Hoyer C, Sartorius A. Clinically favourable effects of ketamine as an anaesthetic for electroconvulsive therapy: a retrospective study. Eur Arch Psychiatry Clin Neurosci. 2011. 10.1007/s00406-011-0205-7 21400226

[31] McDaniel WW, Sahota AK, Vyas BV, Laguerta N, Hategan L, Oswald J (2006). Ketamine appears associated with better word recall than etomidate after a course of 6 electroconvulsive therapies. J ECT.

[32] Okamoto N, Nakai T, Sakamoto K, Nagafusa Y, Higuchi T, Nishikawa T (2010). Rapid antidepressant effect of ketamine anesthesia during electroconvulsive therapy of treatment-resistant depression: comparing ketamine and propofol anesthesia. J ECT.

[33] Rasmussen KG, Kung S, Lapid MI, Oesterle TS, Geske JR, Nuttall GA (2014). A randomized comparison of ketamine versus methohexital anesthesia in electroconvulsive therapy. Psychiatry Res.

[34] Yen T, Khafaja M, Lam N, Crumbacher J, Schrader R, Rask J (2015). Post-Electroconvulsive Therapy Recovery and Reorientation Time With Methohexital and Ketamine: A Randomized, Longitudinal, Crossover Design Trial. J ECT.

[35] Yoosefi A, Sepehri AS, Kargar M, Akhondzadeh S, Sadeghi M, Rafei A (2014). Comparing effects of ketamine and thiopental administration during electroconvulsive therapy in patients with major depressive disorder: a randomized, double-blind study. J ECT.

[36] Loo CK, Katalinic N, Garfield JB, Sainsbury K, Hadzi-Pavlovic D, Mac-Pherson R (2012). Neuropsychological and mood effects of ketamine in electroconvulsive therapy: a randomised controlled trial. J Affect Disord.

[37] McGirr A, Berlim MT, Bond DJ, Neufeld NH, Chan PY, Yatham LN (2015). A systematic review and meta-analysis of randomized controlled trials of adjunctive ketamine in electroconvulsive therapy: efficacy and tolerability. J Psychiatr Res.

[38] Fitzgerald PB, Laird AR, Maller J, Daskalakis ZJ (2008). A meta-analytic study of changes in brain activation in depression. Hum Brain Mapp.

[39] Bench CJ, Friston KJ, Brown RG, Frackowiak RS, Dolan RJ (1993). Regional cerebral blood flow in depression measured by positron emission tomography: the relationship with clinical dimensions. Psychol Med.

[40] Bench CJ, Frackowiak RS, Dolan RJ (1995). Changes in regional cerebral blood flow on recovery from depression. Psychol Med.

[41] Schmidt EZ, Reininghaus B, Enzinger C, Ebner C, Hofmann P, Kapfhammer HP (2008). Changes in brain metabolism after ECT-positron emission tomography in the assessment of changes in glucose metabolism subsequent to electroconvulsive therapy--lessons, limitations and future applications. J Affect Disord.

[42] Nobler MS, Sackeim HA (2008). Neurobiological correlates of the cognitive side effects of electroconvulsive therapy. J ECT.

[43] Sheenan DV, Lecrubier Y, Harnett-Sheehan K, Amorim P, Janavs J, Weiller E (1998). The Mini International Neuropsychiatric Interview (M.I.N.I.): The Development and Validation of a Structured Diagnostic Psychiatric Interview. J Clin Psychiatry.

[44] Wechsler D (2001). Wechsler Test of Adult Reading.

[45] Folstein MF, Folstein SE, McHugh PR (1975). ‘Mini-Mental State’: a practical method for grading the cognitive state of patients for the clinician. J Psychiatr Res.

[46] Royal College of Psychiatrists (2005). The ECT Handbook Second Edition.

[47] Benedict RH, Schretlen D, Groninger L, Brandt J (1998). Hopkins Verbal Learning Test - Revised: Normative data and analysis of inter-form and test-retest reliability. Clin Neuropsychol.

[48] Montgomery SA, Asberg MA (1979). A new depression scale designed to be sensitive to change. Br J Psychiatry.

[49] Murkin JM, Arango M (2009). Near-infrared spectroscopy as an index of brain and tissue oxygenation. Br J Anaesth.

[50] Wolf M, Ferrari M, Quaresima V (2007). Progress of near-infrared spectroscopy and topography for brain and muscle clinical applications. J Biomed Opt.

[51] Greisen G (2006). Is near-infrared spectroscopy living up to its promises?. Semin Fetal Neonatal Med.

[52] Blasi A, Fox S, Everdell N, Volein A, Tucker L, Csibra G (2007). Investigation of depth dependent changes in cerebral haemodynamics during face perception in infants. Phys Med Biol.

[53] Eranti S, Mogg A, Pluck G, Landau S, Purvis R, Brown RG (2007). A randomized, controlled trial with 6-month follow-up of repetitive transcranial magnetic stimulation and electroconvulsive therapy for severe depression. Am J Psychiatry.

[54] Matsuo K, Kato T, Fukuda M, Kato N (2000). Alteration of hemoglobin oxygenation in the frontal region in elderly depressed patients as measured by near-infrared spectroscopy. J Neuropsychiatry Clin Neurosci..

[55] Huppert TJ, Diamond SG, Franceschini MA, Boas DA (2009). HomER: a review of time-series analysis methods for near-infrared spectroscopy of the brain. Appl Opt.

[56] Department of Health (2015). Electro Convulsive Therapy: Survey covering the period from January 2002 to March 2002.

[57] Scottish ECT Accreditation Network. Scottish ECT Accreditation Network Annual Report 2014: A summary of ECT in Scotland for 2013. http://www.sean.org.uk/AuditReport/SEAN-Report-2014-web.pdf. 2014. accessed 27-8-2015.

[58] Porter R, Heenan H, Reeves J (2008). Early effects of electroconvulsive therapy on cognitive function. J ECT.

[59] McElhiney M, Moody B, Sackeim H (1997). The Autobiographical Memory Interview – Short Form.

[60] Benton LA, Hamsher K, Sivan AB (1994). Controlled Oral Word Association Test.

[61] Meador KJ, Moore EE, Nichols ME, Abney OL, Taylor HS, Zamrini EY (1993). The role of cholinergic systems in visuospatial processing and memory. J Clin Exp Neuropsychol.

[62] Wechsler D (1981). Wechsler Adult Intelligence Scale-Revised.

[63] Berman RM, Prudic J, Brakemeier EL, Olfson M, Sackeim HA (2008). Subjective evaluation of the therapeutic and cognitive effects of electroconvulsive therapy. Brain Stimul.

[64] Sobin C, Sackeim HA, Prudic J, Devanand DP, Moody BJ, McElhiney MC (1995). Predictors of retrograde amnesia following ECT. Am J Psychiatry.

[65] Snaith RP, Baugh SJ, Clayden AD, Husain A, Sipple MA (1982). The Clinical Anxiety Scale: an instrument derived from the Hamilton Anxiety Scale. Br J Psychiatry.

[66] Overall JE, Gorman DR (2011). The Brief Psychiatric Rating Scale. Psychol Rep.

[67] Guy W (1976). ECDEU assessment manual for psychopharmacology, revised. US Department of Health, Education and Welfare publication (ADM).

[68] Rush AJ, Trivedi MH, Ibrahim HM, Carmody TJ, Arnow B, Klein DN (2003). The 16-Item Quick Inventory of Depressive Symptomatology (QIDS), clinician rating (QIDS-C), and self-report (QIDS-SR): a psychometric evaluation in patients with chronic major depression. Biol Psychiatry.

[69] The EuroQol Group (2009). EuroQol-a new facility for the measurement of health-related quality of life. Health Policy.

